# Mental Health among Left-Behind Children in Rural China in Relation to Parent-Child Communication

**DOI:** 10.3390/ijerph16101855

**Published:** 2019-05-26

**Authors:** Feng Wang, Leesa Lin, Mingming Xu, Leah Li, Jingjing Lu, Xudong Zhou

**Affiliations:** 1 The Institute of Social and Family Medicine, School of Medicine, Zhejiang University, 866 Yuhangtang Rd., Hangzhou 310058, China; wangfeng1990@zju.edu.cn (F.W.); 3150105441@zju.edu.cn (M.X.); jingjinglu@zju.edu.cn (J.L.); 2 Faculty of Public Health Policy, London School of Hygiene & Tropical Medicine, 15-17 Tavistock PL, Kings Cross, London WC1H9SH, UK; Leesa.Lin@lshtm.ac.uk; 3 Department of Global Health and Social Medicine, Harvard Medical School, 641 Huntington Avenue, Boston, MA 02215, USA; Leesa.Lin@hms.harvard.edu; 4 Population, Policy and Practice Programme, GOS Institute of Child Health, University College London, Guilford St, London WC1N1EH, UK; leah.li@ucl.ac.uk

**Keywords:** left-behind children, mental health, parent-child communication, migration, China

## Abstract

In China, there are an estimated 41 million left-behind children (LBC). The objective of this study was to examine the mental health of current-left-behind children (current-LBC) and previous-left-behind children (previous-LBC) as compared to never-left-behind children (never-LBC), while considering factors like parent-child communication. Children were recruited from schools in rural areas of Anhui province in eastern China. Participants completed a questionnaire focusing on migration status, mental health, and parent-child communication, measured with the validated Strengths and Difficulties Questionnaire (SDQ) and Parent–Adolescent Communication Scale (PACS). Full data were available for 1251 current-, 473 previous-, and 268 never-LBC in Anhui province. After adjusting for all confounding variables, the results showed that both current and previous parental migration was associated with significantly higher mental health difficulties, including aspects of emotional symptoms, conduct problems, hyperactivity, and total difficulties. Additionally, we found that difficulties communicating with parents were strongly associated with the presence of greater total difficulties in children. Parental migration has an independent, long-lasting negative effect on children. Poor parent-child communication is strongly associated with children’s mental health. These results indicate that parent–child communication is important for the development of children, and interventions are needed to improve migrant parents’ understanding and communication skills with their children.

## 1. Introduction

There are over 41 million children aged 18 years or younger who were left behind by their migrant parents in rural China; nearly one in three children in rural areas was left behind [1]. Left-behind children (LBC) are children “under 18 who were left behind at their rural communities while one or both of their parents migrated into cities for work, and who have not lived with them for over six months“ [2]. With the acceleration of urbanization and industrialization over the past four decades, China experienced the largest rural-urban migration in human history. The number of rural–urban migrants is 247 million or 31% of the working population nationally [3]. In China, however, a number of restrictions on rural-urban migration persist—mainly the *hukou* household registration system, a place-based public resource distribution and management system which restricts migrants and their children from accessing education, healthcare, and other public services in the cities to which they migrate [4]. Most migrants are employed in low-paying jobs and live in poor conditions, further discouraging them from bringing their children to the cities to which they migrate [5]. All of these factors exacerbated the LBC phenomenon, making it particularly severe in China.

The problems associated with separating children from parents due to transient employment are not unique to China. Many low- and middle-income countries, such as the Philippines, Vietnam, and Thailand, experience high levels of LBC [6,7,8]. The family systems theory provides a useful framework to understand mental health in children from these migrant families. The theory proposes that parental migration in the family has mixed effects on children’s adjustment through a trade-off between an increase in family income and a decline in parental care [9,10]. For example, while augmented income through remittances may contribute to improved nutrition and general health for children, parental absence may decrease care, stimulation, and communication, leading to the emergence of psychological and behavioral problems. A considerable amount of research was published on the effects of parental migration on LBC. These studies demonstrated that LBC in rural China experience more challenges compared to their counterparts living with parents with respect to loneliness, depression, anxiety [10,11,12,13,14,15,16,17,18,19], and behavioral problems [20,21,22]. However, most studies on LBC’s mental health do not take into account the dynamism and complex nature of the family relationships [23]. For example, some parents of LBC may return to their hometown after migrating for work for an extended period. Previously collected data were limited regarding the differences between current-LBC and previous-LBC [14].

Moreover, less attention is paid to the important family process of parent–child communication, which refers to the interaction between parents and children (including verbal and nonverbal interactions) [24]. Parent-child communication, as an indicator of the strength of the parent–child relationship, is emphasized as an important family factor contributing to children’s development [25]. LBC face the adverse effects of long-term parental absence because of parents’ rural-to-urban migration, which may exert lasting negative effects on family structure and the parent-child bond [26]. Some studies on LBC demonstrated that mental health and behavioral problems are closely related to the quality of their communication with their parents [27]. Despite these studies, the effects of parent-child communication on children’s mental health with regard to different forms of parental migration remain unclear.

The present study seeks to investigate the following: (1) whether children who are currently left behind (current-LBC), who were previously left behind (previous-LBC), and who were never left behind (never-LBC) differ with regard to mental health, and (2) how the quality of parent-child communication affects children’s mental health across current-LBC, previous-LBC, and never-LBC.

## 2. Materials and Methods

The current study was a cross-sectional survey using self-reported questionnaires. Participants were recruited from rural areas of Anhui, a relatively underdeveloped southeastern province in China, which is a feeder location for migrant workers, with around 16 million migrant workers and 4.5 million LBC [2]. Two counties in southeast Anhui Province (Wuwei and Nanling) were included in the study.

### 2.1. Sample

For the ease of sampling, we aimed to select areas where there were large numbers of LBC. To accomplish this, we obtained child population figures from the 2015 National Inter-Census Survey [1]. We then selected two counties in Anhui Province, with high proportions of LBC in rural areas. Two towns were randomly selected from each county, for a total of four towns. Two random schools in each selected township were then included in the study. In each school, all children from Grades 5–8 (mostly including children aged 11 to 17) were invited to participate. Younger children were not included to ensure participants had the literacy level necessary to complete the questionnaire. Considering that the effects of diverse forms of parental absence on children may vary, children who were orphans or came from single-parent families were excluded.

Ethical approval was obtained from Zhejiang University (Project Number ZGL201804-2) and local approvals were obtained from county authorities. Permission to conduct the study in the schools was obtained from the individual head teachers. Before the survey, informed consent was obtained from both the eligible children and their parents or guardians (through a letter sent home). If consent was given on both forms, the student was asked to complete a self-administered questionnaire in their classroom. Given the sensitivity of some of the questions, researchers administered the questionnaire in the classroom without the presence of any teachers or school administrators. Participants were told that there was no compulsion to complete the questionnaire, even after consent was obtained. Anonymity and confidentiality were assured.

### 2.2. Measures

#### 2.2.1. Demographic Characteristics

All participants were asked to provide information on their gender, age, grade, number of siblings, and family economic status in comparison with others in the community (much better off/better off, the same, poorer/much poorer).

To determine the status of parental migration, students were asked to answer two separate questions: “did your father (and mother) migrate into cities for work and does he (and she) no longer live with you for the last six months?” The answers were “no, never”, “yes, previously migrated”, and “yes, currently migrated”. If neither parent migrated, the participant was defined as “never-LBC”; if one or both parents were currently migrated, the participant was defined as “current-LBC”; if not, and if one or both parents previously migrated, the participant was defined as “previous-LBC”.

#### 2.2.2. The Strength and Difficulties Questionnaires (SDQ)

The mental health of children was measured using the Chinese student version of the SDQ, which was widely used and validated in the Chinese context [28,29]. As opposed to the parent or teacher version, the student version of the SDQ is regarded as most suitable for this age group [30]. The SDQ consists of 25 items regarding mental health in five subscales: conduct problems, peer problems, emotional symptoms, pro-social behaviors, and hyperactivity. Each item is scored on a three-point Likert scale (0–2), with zero denoting not sure, and two denoting certainly sure. The score for each subscale is obtained by computing the sum of its five items. All scales but the pro-social subscale were taken together to generate the total difficulties score, with its values ranging from 0 to 40. In all subscales (excluding the pro-social behavior subscale), higher scores mean a lower sense of well-being and more problematic behaviors.

#### 2.2.3. Parent-Child Communication

Parent-child communication was assessed with the Chinese version of the Parent–Adolescent Communication Scale (PACS) [24,31,32]. The scale is a 20-item tool where participants rank certain statements along a five-point continuum from “strongly agree” to “strongly disagree”. The PACS has two subscales: the “open family communication” subscale and the “problems in family communication” subscale. The “open family communication” subscale has ten items, such as “my father/mother tries to understand my point of view” and “I find it easy to discuss problems with my father/mother”. The “problems in family communication” subscale also has ten items, including “I don’t think I can tell my father/mother how I really feel about some things” and “I am sometimes afraid to ask my father/mother for what I want”. The scores for items on the “problems” subscale are reversed in value. Thus, a high score indicates a lack of perceived problems in communication. This conversion provides an additive total scale score; higher scores represent better parent–adolescent communication. The participants were asked to appraise communication with their mother and father separately. The questionnaire was found to have good internal consistency and construct validity in Chinese settings [33,34]. The Cronbach α scores were 0.793 and 0.927 for each subscale in this study.

### 2.3. Statistical Analysis

Firstly, chi-square tests (for categorical variables) or analyses of variance (for continuous variables) were conducted to compare sample characteristics among the three groups of children with different parental migration status. Secondly, ANOVA was performed to compare group differences in terms of PACS and SDQ. A post hoc comparison was conducted using the least significant difference criterion. Finally, multiple linear regression models were employed to examine the associations between mental health outcomes and parental migration status. The baseline model included child demographics (gender, grade, economic status, and number of siblings). The model was further adjusted for parent-adolescent communication quality, including mother–adolescent communication and father-adolescent communication. Regression coefficients and the 95% confidence interval are presented. The interactions between covariates and migration status were also tested. All statistical analyses were performed using SPSS 24.0 (IBM, Armonk, NY, USA) for Windows.

## 3. Results

### 3.1. Socio-Demographic Characteristics 

Completed questionnaires were obtained from a total of 1922 participants, comprising 1251 current-LBC, 473 previous-LBC, and 268 never-LBC. Of the total classroom sample, 27 (1.3%) declined to complete the questionnaire, and another 39 (1.9%) failed to report parental migration status. The demographic characteristics of the participants are shown in Table 1. Overall, there were more male (54.4%) than female (45.6%) respondents, and this did not differ across the three groups. The mean age of previous-LBC was slightly older (mean 13.2, SD 1.2) than the current-LBC and never-LBC groups. Approximately one-third of the children were only children across the three groups. In terms of self-reported comparative socioeconomic status, there were no significant differences among groups.

### 3.2. Comparison of PACS and SDQ Scores 

Key findings from the mean PACS scores for all subscales with comparisons shown by child group are available in Table 2. Except for problems in the communication with father category, there were significant between-group differences in all PACS factors. Pairwise comparisons demonstrated the previous-LBC group had significantly less openness (both with mother and father), more problems (with mother), and lower total scores (both with mother and father) than the never-LBC group. According to post hoc tests, there were no significant differences between the current-LBC and previous-LBC in total or subscale scores, with the exception of total score regarding communication with mothers. Table 2 also shows the mean SDQ scores for all subscales and total difficulties across the three groups of children. The total difficulties score was significantly higher in current-LBC and previous-LBC, as well as the three difficulty subscales (emotional symptoms, conduct problems, and hyperactivity). According to post hoc tests, there were few differences in total or subscale scores between the current-LBC and previous-LBC.

### 3.3. Association with SDQ Subscale Scores 

The regression results of SDQ subscale scores are listed in Table 3. Both current and previous parental absence demonstrated a strong association with higher conduct problems and hyperactivity after adjusting for all covariates. Current-LBC appeared to be more disadvantaged than previous-LBC in terms of emotional symptoms when both were compared to never-LBC. Girls scored higher on emotional difficulties. Older children (Grade 7 and Grade 8) tended to have fewer conduct problems, but greater hyperactivity, compared to younger children (Grade 5 and Grade 6). Having problems in communication with mother or father was positively associated with emotional symptoms, conduct problems, and hyperactivity. Children who experienced higher levels of openness in family communication (with mother or father) were less likely to have hyperactivity problems.

### 3.4. Association with SDQ Total Difficulties Scores 

Table 4 represents the association between parental migration status and the SDQ total difficulties. Both current-LBC and previous-LBC were significantly more likely to have higher total difficulties scores. After adjusting for all confounding variables, the increases in total difficulties score were still significant in current-LBC (B = 1.55, *p* < 0.001) and in previous-LBC (B = 0.80, *p* < 0.05) when compared to never-LBC. Older children (Grade 7 and Grade 8) and children with siblings tended to be more vulnerable, but only in the baseline model (B = 0.69, *p* < 0.01; B = −0.58, *p* < 0.05). Children from poorer families were more likely to have higher total difficulties after adjusting for all covariates (B = 1.03, *p* < 0.05). Problems in communication with mother and/or father showed positive associations with the total difficulties score. Additionally, openness in communication with mother, rather than with father, was strongly linked with better well-being (B = −0.11, *p* < 0.001).

## 4. Discussion

The current study sought to examine the impact of parental migration on the mental health of children by directly comparing current-LBC and previous-LBC. Another contribution of this research is the collection of detailed information about a range of child well-being factors, and how they correlate with family characteristics and dynamic family relationships. We demonstrated that children of migrant parents, including both current-LBC and previous-LBC, are significantly more likely to report higher levels of emotional symptoms, higher levels of conduct problems, and higher levels of hyperactivity than never-LBC. We also observed a significant effect of parent–child communication on children’s mental health. Our findings highlighted a number of important issues and provided some information relevant to developing effective intervention programs.

Firstly, our study suggested that, after controlling for the major confounding variables, both the previous and current experience of long-term separation with migrant parents manifested adverse effects on a child’s mental health, with strong and consistent associations. Consistent with most previous studies, the findings of the current study revealed that parental migration has long-term negative impacts on children’s emotional and behavioral problems [35,36]. Migrant parents’ return will not necessarily reverse the consequences of a prolonged absence in their children’s life. This may be due to a number of possible explanations. As migration represents a change in primary caregiver, a parents’ return might create new challenges in their child’s life [37]. In a previous qualitative study in rural Zhejiang Province, in-depth interviews with 17 migrant parents found that some migrants decide to return home permanently because of their child’s growth or the occurrence of certain worrying events [38]. However, future studies should pay specific attention to the risks faced by previous-LBC.

Secondly, poor parent-adolescent communication is strongly associated with children’s mental health. No significant interaction effects, however, were found between the various forms of parental migration status and parent-adolescent communication. The attachment theory provides a useful framework to understand the mental health of children from these families with poorer parent–adolescent communication [39]. Early experiences with parents are important in developing secure attachment relationships. Experiences of separation and the disruption of parent-adolescent communication may cause the development of insecure attachment relationships in children. According to the attachment theory, children with insecure attachment may show more internalizing problems (e.g., emotional symptoms) and externalizing problems (e.g., conduct problems and hyperactivity) when they are in stressful situations. This implies that migrant parents should focus on improving communication skills and supporting their children when they are working from a distance. Targeted family-based interventions that concentrate on improving parent–adolescent communication should be implemented in feeder communities for migrant workers.

Thirdly, our work demonstrates interesting demographic influences. It is noteworthy that the association with mental health outcomes between gender, age, wealth level, and having siblings differed in different measures and, in some cases, even showed opposite directions. However, more research on this topic needs to be undertaken before the specific mechanism of these correlations is more clearly understood. Pertinent care programs should be developed for children in various socio-demographic groups.

Several limitations need to be considered. Firstly, these findings are limited by the use of a cross-sectional design. Future studies which use a longitudinal design are needed to explore these issues. Secondly, results from two counties from one underdeveloped province in China must be extrapolated with caution. Thirdly, the use of a self-reported continuum of mental health, instead of a clinical diagnosis, limits our conclusions. Caution should be taken when generalizing the current results to clinical conditions. Fourthly, the current study only examined a limited range of potential determinants; some issues that were not addressed in this study were children’s relationship with their caretakers (i.e., grandparents, etc.) and related factors (i.e., family social capital, etc.).

Notwithstanding these limitations, the findings of this research strongly suggest that parental migration is detrimental to the mental health of LBC, independent of family-related factors and parent–adolescent communication. In addition, our research highlights the lasting negative impacts on children of left-behind experiences, which were often neglected in previous studies. Given our results, the observance of a relative decrease in LBC is encouraging. The number of LBC decreased dramatically over the last five years, decreasing from 61 million to 41 million between 2010 and 2015 [1]. This aligns with the Chinese government’s policies to provide better care and protection to LBC. However, at the current level of 41 million children, the growing evidence of the negative consequences of parental migration on children cannot be ignored, while it is clearly undesirable and unfeasible to prevent parents from engaging in work-related migration. We must determine what can be done to support the millions of children with high rates of internalizing problems and externalizing problems. Given the significant correlation between parent–adolescent communication and child mental health, local governments and migrant-sending communities should make efforts to train migrant parents to improve their understanding of the importance of parent–child communication and to focus on improving their communication skills with their left-behind children. Furthermore, local communities should provide more support for the most vulnerable children.

## 5. Conclusions

In conclusion, the very common phenomenon of LBC will continue in China for the foreseeable future and the negative effects of parental migration on children will also persist. This work explored the differences between children who are currently left behind and children who were previously left behind, thus extending existing knowledge on previous-LBC, who are often neglected in academic studies. Our findings contribute to the literature and have important implications for the development of interventions to improve the mental health of LBC in rural China. When children are left behind, migrant parents should be encouraged to maintain regular and positive communication with their children.

## Figures and Tables

**Table 1:** Sample characteristics by parental migration status, *n* (%). LBC—left-behind children.

Variables	Current-LBC	Previous-LBC	Never-LBC	F or χ^2^	*p-*Value
Gender				1.08	0.584
Male	678 (54.9)	257 (55.0)	137 (51.5)		
Female	558 (45.1)	210 (45.0)	129 (48.5)		
Age, mean (SD)	13.1 (1.2)	13.2 (1.2)	13.0 (1.2)	4.25	0.014
Grade				7.76	0.021
Grade 5 Grade 6	538 (43.0)	184 (38.9)	132 (49.4)		
Grade 7 Grade 8	712 (57.0)	289 (61.1)	135 (50.6)		
Income level				5.94	0.204
Much better off/better off	317 (25.5)	120 (25.7)	85 (32.0)		
The same	818 (65.9)	306 (65.5)	165 (62.0)		
Poorer/much poorer	106 (8.5)	41 (8.8)	16 (6.0)		
Any siblings				3.69	0.158
Yes	820 (65.5)	314 (66.4)	192 (71.6)		
No	431 (34.5)	159 (33.6)	76 (28.4)		

**Table 2:** Comparisons among current-LBC, previous-LBC, and never-LBC regarding the Parent-Adolescent Communication Scale (PACS) and the Strength and Difficulties Questionnaire (SDQ) scores; mean (SD).

Variables	Current-LBC (1)	Previous-LBC (2)	Never-LBC (3)	F	PC ^&^
Mother-adolescent communication					
Openness subscale	28.4 (5.8)	27.7 (5.7)	28.6 (5.4)	3.12 *	(2,3)
Problem subscale	23.0 (4.9)	23.6 (4.9)	22.7 (4.5)	3.90 *	(2,3)
Total scale	55.4 (9.7)	54.1 (9.2)	56.0 (8.9)	4.50 *	(1,2) (2,3)
Father-adolescent communication					
Openness subscale	28.1 (6.1)	27.3 (6.4)	28.6 (6.1)	4.46 *	(2,3)
Problem subscale	21.9 (5.1)	22.4 (5.3)	21.5 (5.1)	2.89	
Total scale	56.1 (10.2)	54.9 (10.5)	57.3 (10.1)	5.06 **	(2,3)
SDQ					
Emotional symptoms	3.6 (2.2)	3.4 (2.1)	3.0 (2.1)	7.47 **	(1,3) (2,3)
Conduct problems	2.5 (1.6)	2.5 (1.6)	2.1 (1.6)	4.80 **	(1,3) (2,3)
Hyperactivity	4.1 (2.1)	4.0 (2.1)	3.5 (2.1)	8.54 ***	(1,3) (2,3)
Peer problems	2.7 (1.7)	2.6 (1.6)	2.5 (1.6)	1.14	
Total difficulties score	12.7 (5.4)	12.5 (5.2)	11.1 (5.1)	10.02 ***	(1,3) (2,3)
Pro-social	6.9 (2.0)	7.0 (2.0)	7.1 (1.9)	2.00	

* *p* < 0.05, ** *p* < 0.01, *** *p* < 0.001; PC ^&^ indicates the significance of pairwise comparisons in the post hoc analysis.

**Table 3:** Regression analysis for SDQ (emotional symptoms, conduct problems, hyperactivity) by type of child, parent-adolescent communication, and demographic characteristic.

Variables	Emotional Symptoms ^#^	Conduct Problems ^$^	Hyperactivity ^&^
Parental migration status (reference: never-LBC)			
current-LBC	0.60 (0.32, 0.87) ***	0.31 (0.09, 0.52) **	0.53 (0.27, 0.80) ***
previous-LBC	0.29 (−0.02, 0.61)	0.25 (0.01, 0.49) *	0.33 (0.02, 0.63) *
Gender (reference: male)			
Female	0.64 (0.46, 0.83) ***	−0.07 (−0.21, 0.08)	0.10 (−0.08, 0.28)
Grade (reference: Grade 5, Grade 6)			
Grade 7, Grade 8	−0.16 (−0.35, 0.03)	−0.15 (−0.30, 0.01) *	0.34 (0.16, 0.52) ***
Income level (reference: much better/better/the same)			
Poorer/much poorer	0.28 (−0.05, 0.61)	0.15 (−0.11, 0.40)	0.11 (−0.21, 0.43)
Any siblings (reference: yes)			
No	−0.14 (−0.34, 0.05)	−0.08 (−0.23, 0.07)	−0.20 (−0.38, −0.01) *
Mother-adolescent communication			
Openness subscale	−0.02 (−0.04, 0.01)	−0.01 (−0.03, 0.01)	−0.05 (−0.07, −0.03) ***
Problem subscale	0.11 (0.08, 0.14) ***	0.08 (0.06, 0.11) ***	0.09 (0.06, 0.12) ***
Father-adolescent communication			
Openness subscale	−0.02 (−0.04, 0.01)	0.01 (−0.01, 0.02)	−0.03 (−0.05, −0.01) *
Problem subscale	0.04 (0.01, 0.06) **	0.04 (0.02, 0.06) **	0.04 (0.01, 0.07) **

* *p* < 0.05, ** *p* < 0.01, *** *p* < 0.001; ^#^: adjusted *R^2^* = 0.163; ^$^: adjusted *R^2^* = 0.120; ^&^: adjusted *R^2^* = 0.201.

**Table 4:** Total difficulties on parental migration status with and without adjustment for parent–adolescent communication.

Variables	Baseline Model ^#^	Adjusted Model ^$^
Parental migration status (reference: never-LBC)		
current-LBC	1.61 (0.90, 2.32) ***	1.55 (0.90, 2.20) ***
previous-LBC	1.37 (0.56, 2.18) **	0.80 (0.06, 1.55) *
Gender (reference: male)		
Female	0.19 (−0.29, 0.67)	0.42 (−0.02, 0.86)
Grade (reference: Grade 5, Grade 6)		
Grade 7, Grade 8	0.69 (0.21, 1.16) **	−0.43 (−0.88, 0.02)
Income level (reference: much better/better/the same)		
Poorer/much poorer	1.65 (0.80, 2.51) ***	1.03 (0.25, 1.80) *
Any siblings (reference: yes)		
No	−0.58 (−1.10, −0.08) *	−0.38 (−0.84, 0.09)
Mother-adolescent communication		
Openness subscale		−0.11 (−0.16, −0.05) ***
Problem subscale		0.29 (0.22, 0.36) ***
Father-adolescent communication		
Openness subscale		−0.04 (−0.10, 0.01)
Problem subscale		0.15 (0.09, 0.22) ***

* *p* < 0.05, ** *p* < 0.01, *** *p* < 0.001; ^#^: adjusted *R^2^* = 0.022; ^$^: adjusted *R^2^* = 0.244.

## References

[1] Lv L., Yan F., Duan C., Cheng M. (2018). Changing patterns and development challenge of child population in China. Popul. Res..

[2] Duan C., Lv L., Guo J., Wang Z. (2013). Survival and development of left-behind children in rural China: Based on the analysis of sixth census data. Popul. J..

[3] National Bureau of Statistics of China (2016). Statistical Communiqué of the People’s Republic of China on the 2014 National Economic and Social Development.

[4] Peng X. (2011). China’s demographic history and future challenges. Science.

[5] Chen X., Wang L., Wang Z. (2009). Shyness-sensitivity and social, school, and psychological adjustment in rural migrant and urban children in China. Child Dev..

[6] Antman F.M. (2011). International migration and gender discrimination among children left behind. Am. Econ. Rev..

[7] Jordan L.P., Graham E. (2012). Resilience and well-being among children of migrant parents in south-east Asia. Child Dev..

[8] Srneekens C., Stroebe M.S., Abakoumkin G. (2012). The impact of migratory separation from parents on the health of adolescents in the Philippines. Soc. Sci. Med..

[9] Lu Y. (2012). Household migration, social support, and psychosocial health: The perspective from migrant-sending areas. Soc. Sci. Med..

[10] Wang L., Mesman J. (2015). Child development in the face of rural-to-urban migration in China: A meta-analytic review. Perspect. Psychol. Sci..

[11] Liu Z., Li X., Ge X. (2009). Left too early: The effects of age at separation from parents on Chinese rural children’s symptoms of anxiety and depression. Am. J. Public Health.

[12] Wang L., Feng Z., Yang G., Yang Y., Dai Q., Hu C. (2015). The epidemiological characteristics of depressive symptoms in the left-behind children and adolescents of Chongqing in China. J. Affect. Disord..

[13] Wen M., Lin D. (2012). Child development in rural China: Children left behind by their migrant parents and children of nonmigrant families. Child Dev..

[14] Wu Q., Lu D., Kang M. (2015). Social capital and the mental health of children in rural China with different experiences of parental migration. Soc. Sci. Med..

[15] Fan F., Su L., Gill M.K., Birmaher B. (2010). Emotional and behavioral problems of Chinese left-behind children: A preliminary study. Soc. Psychiatry Psychiatr. Epidemiol..

[16] Shen M., Gao J., Liang Z., Wang Y., Du Y., Stallones L. (2015). Parental migration patterns and risk of depression and anxiety disorder among rural children aged 10–18 years in China: A cross-sectional study. BMJ Open.

[17] Jia Z., Shi L., Cao Y., Delancey J., Tian W. (2010). Health-related quality of life of “left-behind children”: A cross-sectional survey in rural China. Qual. Life Res..

[18] Huang Y., Zhong X., Li Q., Xu D., Zhang X., Feng C. (2015). Health-related quality of life of the rural-China left-behind children or adolescents and influential factors: A cross-sectional study. Health Qual. Life Outcomes.

[19] He B., Fan J., Liu N., Li H., Wang Y., Williams J. (2012). Depression risk of ‘left-behind children’ in rural China. Psychiatry Res..

[20] Gao Y., Li L., Chan E.Y.Y., Lau J., Griffiths S.M. (2013). Parental migration, self-efficacy and cigarette smoking among rural adolescents in south China. PLoS ONE.

[21] Qu G.B., Wu W., Wang L.L., Tang X., Sun Y.H., Li J. (2018). Systematic review and meta-analysis found higher levels of behavioural problems in male left-behind children aged 6–11 years. Acta Paediatr..

[22] Gao Y., Li L.P., Kim J.H., Congdon N., Lau J., Griffiths S. (2010). The impact of parental migration on health status and health behaviours among left behind adolescent school children in China. BMC Public Health.

[23] Carling J.R., Menjívar C., Schmalzbauer L. (2012). Central themes in the study of transnational parenthood. J. Ethn. Migr. Stud..

[24] Barnes H.L., Olson D.H. (1985). Parent-adolescent communication and the circumplex model. Child Dev..

[25] Formoso D., Gonzales N.A., Aiken L.S. (2000). Family conflict and children’s internalizing and externalizing behavior: Protective factors. Am. J. Community Psychol..

[26] Su S., Li X., Lin D., Xu X., Zhu M. (2013). Psychological adjustment among left-behind children in rural China: The role of parental migration and parent-child communication. Child Care Health Dev..

[27] McCarty C.A., Zimmerman F.J., Digiuseppe D.L., Christakis D.A. (2005). Parental emotional support and subsequent internalizing and externalizing problems among children. J. Dev. Behav. Pediatr..

[28] Goodman R. (1997). The strengths and difficulties questionnaire: A research note. J. Child Psychol. Psychiatry.

[29] Kou J., Du Y., Xia L. (2007). Formulation of children strength and difficulties questionnaire (the edition for students) for Shanghai norm. China J. Health Psychol..

[30] Mazzucato V., Cebotari V., Veale A., White A., Grassi M., Vivet J. (2015). International parental migration and the psychological well-being of children in Ghana, Nigeria, and Angola. Soc. Sci. Med..

[31] Lanz M., Iafrate R., Rosnati R., Scabini E. (1999). Parent-child communication and adolescent self-esteem in separated, intercountry adoptive and intact non-adoptive families. J. Adolesc..

[32] Shek D.T. (2000). Differences between fathers and mothers in the treatment of, and relationship with, their teenage children: Perceptions of Chinese adolescents. Adolescence.

[33] Ying L., Ma F., Huang H., Guo X., Chen C., Xu F. (2015). Parental monitoring, parent-adolescent communication, and adolescents’ trust in their parents in China. PLoS ONE.

[34] Xia Y.R., Xie X., Zhou Z., Defrain J., Defrain J., Combs R. (2004). Chinese adolescents’ decision-making, parent-adolescent communication and relationships. Marriage Fam. Rev..

[35] Zhao C., Wang F., Li L., Zhou X., Hesketh T. (2017). Long-term impacts of parental migration on Chinese children’s psychosocial well-being: Mitigating and exacerbating factors. Soc. Psychiatry Psychiatr. Epidemiol..

[36] Wang F., Zhou X., Hesketh T. (2017). Psychological adjustment and behaviours in children of migrant workers in China. Child Care Health Dev..

[37] Pottinger A.M. (2005). Children’s experience of loss by parental migration in inner-city Jamaica. Am. J. Orthopsychiatr..

[38] Zhao C., Wang F., Zhou X., Jiang M., Hesketh T. (2018). Impact of parental migration on psychosocial well-being of children left behind: A qualitative study in rural China. Int. J. Equity Health.

[39] Allen J.P., Cassidy J., Shaver P.R. (2008). The attachment system in adolescence. Handbook of Attachment: Theory, Research and Clinical Applications.

